# New York State Veterinary Medical Association

**Published:** 1896-11

**Authors:** 


### NEW YORK STATE VETERINARY MEDICAL ASSOCIATION.

(Continued from page 748.)

The reports of the county secretaries were called up in their regular order by counties. Reports of several county secretaries were made, all of which tended to show the status of the profession in these counties, and also the nature of the work as it came in contact with several boards of health. The discussions were exceedingly interesting, the character of the work being such that justifies the employment of veterinary surgeons on all boards of health throughout the State, as the reports all showed that a great work was being done in protecting innocent consumers of flesh- and dairy-products against unwholesome and infectious food-products. When Kings County was called up the report submitted by Dr. George H. Berns brought out a very interesting and instructive discussion on glanders. The report is as follows:

"In presenting my report as County Secretary for the county of Kings, I am especially indebted to Dr. E. B. Ackerman, veterinary inspector of Brooklyn department of health; Dr. William H. Pendry, dairy inspector, Brooklyn department of health; Dr. E. L. Volgenau, U. S. inspector under Bureau of Animal Industry, stationed at Brooklyn; and superintendent Frank O. Clarke, of the American Society for the Prevention of Cruelty to Animals, for having kindly furnished data from their various departments.

"According to the police census taken some five or six months ago, there are 31,850 horses and about 3000 milch cows in the county. The general health of this number of animals has been exceptionally good during the last year, and were it not for a rather violent outbreak of glanders in the early part of the season, and the extremely hot spell in the middle of August, I am satisfied that the mortality would have been much lower than the average.

"Influenza existed to some extent early in the spring and presented itself in a rather violent form; but was chiefly confined to sale-stables and green stock in general.

"Infectious pneumonia broke out in the government stables at Fort Hamilton in the early spring, and out of about 60 horses 18 were taken with the disease, 15 of which made good recoveries in two or three weeks' time, and 3 while convalescing from pneumonia developed rheumatism, of which 2 recovered fully within a week or two, and 1 remained badly crippled.

"Osteoporosis continues to crop up in all classes of horses, but chiefly in comparatively young animals kept in old and badly-ventilated stables, generally situated on the ground-floor, and without any drainage; about 15 well-marked cases came under my observation, some of which were destroyed and others were disposed of by their owners upon my advice; 2 cases so disposed of are reported as convalescing in other locations and stables.

"Purpura hemorrhagica, pleuro-pneumonia, azoturia, cerebro-spinal meningitis, and colics carried off the usual number.

"Metastatic laminitis following acute indigestion, pneumonia, or influenza, was unusually common this spring, and left quite a number of my patients in such a condition as to render destruction of life advisable.

"The extreme heat of last summer caused very heavy losses in Brooklyn; and dead horses were found on every street, owing to inability of the contractor to remove them fast enough.

"Dr. Ackerman states that of 258 cases reported to the department of health as glandered, farcied, or suspicious, 152 were found to be affected, and accordingly condemned and destroyed. In my own practice probably 30 cases came under observation. The mallein-test was employed in all cases where the symptoms were not sufficiently developed to warrant positive conclusions, and in two stables where glanders had existed for a long time and broke out periodically all animals were tested; those showing a reaction of two degrees or over, and the typical swelling at point of inoculation, were destroyed, and in probably 12 cases where no external symptoms of any description couid be detected post-mortem examinations were made and lesions indicative of glanders were found in every case; yet seven horses which had been among a lot of glandered animals for months, and reacted on the mallein-test from three to five degrees, but were otherwise apparently in good health and showed positively not the slightest indication of glanders that could be detected on physical examination, were allowed to live, and have been carefully watched since last March or April, when the mallein injections were made. At this writing, which is six months after the test, five of these animals appear to be in good health and are doing their ordinary work; one was destroyed as glandered some three months ago, and one has been quarantined as a suspicious subject for the last two months. Is it possible that the germs of glanders are lying dormant all these months in those five horses that are to work? Or did the mallein injection have a curative effect? Or is mallein not altogether reliable as a means of diagnosis?

"Dr. Wm. H. Pendry, in his official report to the department of health states that less than 3 per cent. of the 3000 milch cows in Kings County are affected with tuberculosis, as far as can be detected by physical examination, but qualifies this by saying that most all the cows are of the common hardy variety, frequently changed and retained by their owners only as long as they milk well, and, failing in that, are fattened for slaughter. He speaks of tuberculin as a most reliable diagnostic agent for tuberculosis, and advocates in the strongest terms that this test be applied to all dairy-herds.

"Dr. E. L. Volgenau, United States inspector of the Bureau of Animal Industry, examines all cattle killed at the Hudson Avenue abattoirs. He reports that of 2720 head of fat cows and steers examined by him, 12 were affected with generalized tuberculosis in advanced stages, and a very much larger number with localized tubercular lesions. He concludes that fully,

1 per cent. of all the cattle killed in these abattoirs since December 1, 1895, were tuberculous. He found this disease mostly in fattened cows from local dairies, while actinomycosis was frequently met with in western steers.

"I am pleased to be able to state that the veterinary profession in Kings County is rapidly gaining recognition, confidence, and support from owners of live-stock, the public at large, and our city government. Seventeen years ago, when there was a very much larger number of dairy cattle in the county, and perhaps fully as many horses as they are now (owing to the introduction of the trolley system), not more than four or five regular graduated veterinarians, a few self-educated practitioners, and a dozen or two of ignorant, loud-mouthed empirics found employment in our county, where to-day there are eighty-one regular graduates in veterinary medicine registered in the County Clerk's office, and I believe I am safe in saying that at least 95 per cent. are not only gaining a comfortable livelihood by their practice of the profession, but are looked upon and treated by the community in which they move as scientific and skilful men and respected citizens. Of the thirty-four non-graduated who have registered by affidavit only a very small number are engaged in actual practice, some few of whom are elderly men who educated themselves when there were no veterinary colleges in this county, and by years of study and experience have gained sufficient knowledge and skill to become successful practitioners and control to this day a respectable clientage; the rest are made up of overbearing stable-foremen, conceited horseshoers, unscrupulous horse-cappers, travelling so-called veterinary dentists, and patent-medicine vendors, etc., who took advantage of the laws of 1883 and registered on their affidavits that they had practised for three years; but very few of this latter class remain in practice for Kings County. They were gradually forced from our ranks for a want of support; many have taken down their shingles and left for parts unknown, while others follow other occupations. The city government employs six veterinarians in professional and official capacities, four of whom are connected with the department of health as veterinary inspectors, one as veterinarian to the police department, and one as veterinarian to the fire department.

"In conclusion, I cannot refrain from referring to the excellent work done by the American Society for the Prevention of Cruelty to Animals in this city. It maintains a well-equipped office, consisting of an inspector, four patrolmen, one large ambulance for the removal of horses, and two small and exceedingly well-appointed ambulances for the removal of homeless or, to the owner, objectionable cats and dogs. A summary of the work in this city for 1895 shows: Cases prosecuted in the courts, 952; disabled animals temporarily suspended from labor, 3403; horses and mules disabled and past recovery humanely destroyed, 2987; homeless or disabled small animals humanely destroyed, 46,898; disabled horses removed from streets to veterinary infirmaries or owners' stables, 576; complaints received and investigated, 21,690. These figures show that the officers have not been idle, and that a vast amount of suffering among animals has been prevented by them."

In the discussion that followed, Prof. Law, of Cornell University, said that he thought that slight cases of glanders are not only amenable to treatment but curable, even in our latitude and atmosphere, and on the western plains at high altitudes altogether so. Dr. W. L. Williams, of Cornell, spoke of the prevalence of glanders in Russia and other parts of Europe, and finally of the disease on the ranges in Montana, where glanders exists to some extent, and is not only a curable disease, but that mallein treatment to a large degree produces such results, and that upon the plains the disease is not looked upon with that degree of horror as in the East. Prof. Law, speaking a second time, cited a case of glanders in New York City which was treated, making a complete recovery, a similar case in Ithaca, and numerous cases in Wyoming. Dr. Joseph Hughes, of Chicago, contended that glanders should not be treated, but all affected animals destroyed. Dr. John Wende, of Buffalo, said that he saw three colts with glanders and all made a complete recovery. Dr. W. H. Pendry, of Brooklyn, believed that veterinarians should not advocate the treatment of glanders. Dr. Wilson Huff, of Rome, asked to hear from Dr. Peters, of Nebraska, who believed that glanders is curable on the plains, but that treatment should not be attempted in cities, as the disease is best treated in the open air and on pasture.

(To be continued.)

